# Effect of Tongkat Ali on stress hormones and psychological mood state in moderately stressed subjects

**DOI:** 10.1186/1550-2783-10-28

**Published:** 2013-05-26

**Authors:** Shawn M Talbott, Julie A Talbott, Annie George, Mike Pugh

**Affiliations:** 1 SupplementWatch, 648 Rocky, Knoll Draper, UT 84020, USA; 2 Biotropics Malaysia Berhad, Lot 21, Jalan U1/19, Section U1, Hicom-Glenmarie Industrial Park, 40150 Shah Alam, Selangor, Malaysia; 3 MonaVie, 10855 S River Front Parkway, South Jordan, UT 84095, USA

**Keywords:** Testosterone, Cortisol, Stress, Vigor, Tongkat ali, Eurycoma, Mood

## Abstract

**Background:**

*Eurycoma longifolia* is a medicinal plant commonly called tongkat ali (TA) and “Malaysian ginseng.” TA roots are a traditional “anti-aging” remedy and modern supplements are intended to improve libido, energy, sports performance and weight loss. Previous studies have shown properly-standardized TA to stimulate release of free testosterone, improve sex drive, reduce fatigue, and improve well-being.

**Methods:**

We assessed stress hormones and mood state in 63 subjects (32 men and 31 women) screened for moderate stress and supplemented with a standardized hot-water extract of TA root (TA) or Placebo (PL) for 4 weeks. Analysis of variance (ANOVA) with significance set at p < 0.05 was used to determine differences between groups.

**Results:**

Significant improvements were found in the TA group for Tension (−11%), Anger (−12%), and Confusion (−15%). Stress hormone profile (salivary cortisol and testosterone) was significantly improved by TA supplementation, with reduced cortisol exposure (−16%) and increased testosterone status (+37%).

**Conclusion:**

These results indicate that daily supplementation with tongkat ali root extract improves stress hormone profile and certain mood state parameters, suggesting that this “ancient” remedy may be an effective approach to shielding the body from the detrimental effects of “modern” chronic stress, which may include general day-to-day stress, as well as the stress of dieting, sleep deprivation, and exercise training.

## Background

*Eurycoma longifolia* is an herbal medicinal plant found in South East Asia (Malaysia, Vietnam, Java, Sumatra, Thailand). In Malaysia, it is commonly called tongkat ali and has a range of medicinal properties as a general health tonic, including improvement in physical and mental energy levels and overall quality of life [1,2]. The roots of tongkat ali, often called “Malaysian ginseng”, are used as an adaptogen and as a traditional “anti-aging” remedy to help older individuals adapt to the reduced energy, mood, and libido that often comes with age [3-7]. In modern dietary supplements, tongkat ali can be found in a variety of products intended to improve libido and energy, restore hormonal balance (cortisol/testosterone levels) and enhance both sports performance and weight loss. The objective of this study was to evaluate the effects of tongkat ali extract on stress hormone balance (cortisol/testosterone) and psychological mood state in moderately stressed subjects.

In both men and women, testosterone levels peak between 25 to 30 years of age - and thereafter drop approximately 1-2% annually [8,9]. At the age of 60, testosterone levels are typically only 40-50% of youthful levels and may be lower due to stress and related lifestyle issues such as diet, exercise, and sleep patterns [10,11]. The benefits of maintaining a youthful testosterone levels are many, including increased muscle mass and reduced body fat, high psychological vigor (mental/physical energy), and improved general well-being [12,13].

Eurycoma contains a group of small peptides referred to as “eurypeptides” that are known to have effects in improving energy status and sex drive in studies of rodents [14-16]. The effects of tongkat ali in restoring normal testosterone levels appears to be less due to actually “stimulating” testosterone synthesis, but rather by increasing the release rate of “free” testosterone from its binding hormone, sex-hormone-binding-globulin (SHBG) [17,18]. In this way, eurycoma may be considered not so much a testosterone “booster” (such as an anabolic steroid), but rather a “maintainer” of normal testosterone levels and a “restorer” of normal testosterone levels (from “low” back “up” to normal ranges) [19]. This would make eurycoma particularly beneficial for individuals with sub-normal testosterone levels, including those who are dieting for weight loss, middle-aged individuals suffering with fatigue or depression, and intensely training athletes who may be at risk for overtraining [20,21].

### Traditional use

Decoctions of tongkat ali roots have been used for centuries in Malaysia and Southeast Asia as an aphrodisiac for loss of sexual desire and impotence, as well as to treat a range of ailments including post-partum depression, malaria, high blood pressure, and fatigue [22].

Tongkat ali has been referred to as Malaysia’s “home-grown Viagra” [4] with the Malaysian government investing considerable effort to license, develop, and sustain research into the potential health benefits of *Eurycoma longifolia* through a variety of governmental organizations, including the Forest Research Institute of Malaysia (FIRM) [22].

### Modern extracts

Numerous commercial tongkat ali supplements claim “extract ratios” from 1:20 to 1:200 without any information about bioactive constituents, extraction methodology (e.g. ethanol *versus* water), or extract purity. Alcohol extracts of eurycoma have been studied in mice for antimalarial effects of concentrated eurycomalactone [23] but also exhibit toxic effects at high doses (LD50 at 2.6 g/kg), which would preclude safe use in humans as a long-term dietary supplement [24,25]. In contrast, hot-water root extracts standardized for known bioactive components (1% eurycomanone, 22% protein, 30% polysaccharides, 35% glycosaponin) have been demonstrated to be extremely safe at high doses and for long-term consumption [26-28].

Properly standardized hot-water extracts [2,26,29] have a distinctly bitter taste due to the presence of quassinoids, which are recognized as some of the bitterest compounds in nature [30,31]. Tongkat ali extracts that do not taste bitter are either not true *Eurycoma longifolia* root (there are many commercial examples of “fake” tongkat ali extracts) or are sub-potent in terms of bioactive constituents, and thus would also be expected to have low efficacy. Because of tongkat ali’s reputation for libido benefits, there are several examples of dietary supplements labeled as *Eurycoma longifolia*, but containing none of the actual root, and instead being “spiked” with prescription erectile dysfunction drugs including tadalafil/Cialis, sildenafil/Viagra, and vardenafil/Levitra [4, personal communication].

### Laboratory and animal research

Bhat and Karim [1] conducted an ethnobotanical and pharmacological review on tongkat ali, noting that laboratory research such as cell assay studies offer possible mechanistic support for the myriad traditional uses of tongkat ali, including aphrodisiac [32], antimalarial [33], antimicrobial [34], anti-cancer [35], and anti-diabetic effects [36].

Numerous rodent studies exist demonstrating reduced anxiety and improved sexual performance following tongkat ali feeding [37-40], with such effects thought to be due to a restoration of normal testosterone levels. Eurycoma’s anxiolytic effects have been demonstrated in a variety of behavioral tests, including elevated plus-maze, open field, and anti-fighting, suggesting an equivalent anti-anxiety effect to diazepam as a positive control [37].

Animal studies have shown that many of the effects of the extract are mediated by its glycoprotein components [14]. The mechanism of action of the bioactive complex polypeptides (“eurypeptides” with 36 amino acids) has been shown to activate the CYP17 enzyme (17 alpha-hydroxylase and 17,20 lyase) to enhance the metabolism of pregnenolone and progesterone to yield more DHEA (dehydroepiandrosterone) and androstenedione, respectively [29]. This glycoprotein water-soluble extract of *Eurycoma longifolia* has been shown to deliver anti-aging and anti-stress benefits subsequent to its testosterone-balancing effects [41,42].

Oral toxicity studies (Wistar rats) have determined the LD50 of tongkat ali root extract as 2,000 mg/kg body weight (acute) and the NOAEL (no observed adverse effect level) as greater than 1,000 mg/kg body weight (28-day sub-acute feeding), resulting in a classification as Category 5 (extremely safe) according to the United Nations Globally Harmonized System of Classification and Labeling of Chemicals (GHS).

In addition to the very high safety profile demonstrated in the rodent toxicity studies, there are no reported adverse side effects in human studies of tali supplementation. For example, one 2-month human supplementation trial [27] of twenty healthy males (age range 38–58), found high doses of *Eurycoma longifolia* extract (600 mg/day) to have no influence on blood profiles (hemoglobin, RBC, WBC, etc.) or any deleterious effects on measures of liver or renal function. Typical dosage recommendations, based on traditional use and on the available scientific evidence in humans, including dieters and athletes, call for 50-200 mg/day of a water-extracted tongkat ali root standardized to 22% eurypeptides.

### Human supplementation trials

Based on a long history of traditional use and confirmation of biological activity via cell culture and animal feeding studies, several human supplementation studies have been conducted to evaluate the potential benefits of tongkat ali for sexual function, exercise performance, weight loss, and vigor (mental/physical energy).

Importantly, all of the human trials have used the same water-extracted and standardized eurycoma root for which a patent has been issued jointly to the Government of Malaysia and the Massachusetts Institute of Technology (United States Patent #7,132,117) [29]. The patent discloses a process whereby *Eurycoma longifolia* roots undergo an aqueous extraction combined with HPLC and size-exclusion chromatography to yield a bioactive peptide fraction (a 4300 dalton glycopeptide with 36 amino acids) that is responsible for its effects in maintaining testosterone levels. The bioactive fraction of *Eurycoma longifolia* root delivers a demonstrated ability to improve testosterone levels [41], increase muscle size and strength [43,44], improve overall well-being [45,46], accelerate recovery from exercise [47] enhance weight loss [48,49], reduce stress [50], and reduce symptoms of fatigue [51-53].

Based on it’s long history of traditional medicinal use as an “anti-aging” remedy and the series of animal and human supplementation studies investigating it’s use as a physical and mental performance aid, we undertook a study of the effects of tongkat ali root extract supplementation in moderately stressed subjects. Our hypothesis was that tongkat ali supplementation may influence anabolic/catabolic stress hormone balance and mood state parameters in a group of volunteers with moderate stress levels.

## Methods

All procedures, measurements, and informed consent processes were reviewed and approved by an external third-party review board (Aspire IRB; Santee, CA).

Subjects were recruited in and around Salt Lake City, Utah via flyers asking for volunteers with “moderate stress levels”. We screened approximately 75 subjects for moderate levels of psychological stress. Our intention was to complete the study with 60 subjects (30 subjects per treatment group). We used a screening survey that we have used in past studies of stress/mood to identify individuals with moderately elevated levels of perceived stress [19,21,47-50]. Subjects scoring 6 or greater on this screening survey indicated eligibility for enrollment into the supplementation study (a score of 6–10 indicates moderate stress).

Sixty-four (64) subjects (32 men and 32 women) were randomized to receive tongkat ali (TA; 200 mg/day of Physta™, Biotropics Malaysia Berhad; 32 subjects) or look-alike placebo (PL; 32 subjects) for 4 weeks. The 4-week duration was selected as more representative of persistent changes in mood state that may result from superior hormone balance, as opposed to short-term changes in emotions that may be more closely linked with stressors of daily living.

At Baseline (week 0) and Post-supplementation (week 4), we assessed Mood State and Hormone Profile as our primary outcome measurements. Secondary measurements were made of liver enzymes (ALT; alanine aminotransferase and AST; aspartate aminotransferase; Alere Cholestech, Waltham, MA), body weight, and body fat percentage (Tanita; TBF-300A, Arlington Heights, IL).

Mood State (Vigor, Depression, Anger, Confusion, Fatigue, and Anxiety) was assessed using the validated Profile of Mood States (POMS) survey. Hormone profile (cortisol and testosterone) was assessed in saliva samples collected at three time points during each collection day (morning, afternoon, and evening). Saliva samples were analyzed for free cortisol and free testosterone by enzyme immunoassay (Salimetrics; State College, PA).

Results were analyzed by one-way analysis of variance (ANOVA) with significance set at p < 0.05.

Sixty-three subjects (32 men and 31 women) completed the study, with one woman in the supplement group lost to follow up (did not return final samples).

## Results

Three subjects reported feeling unusually fatigued during the first two weeks of the study (two subjects in the TA group and 1 subject in the placebo group). There were no other adverse events or side effects reported.

Over the course of the supplementation period, there were no significant changes in markers of liver function (AST/ALT), body weight or body fat percentage.

**Figure 1:**
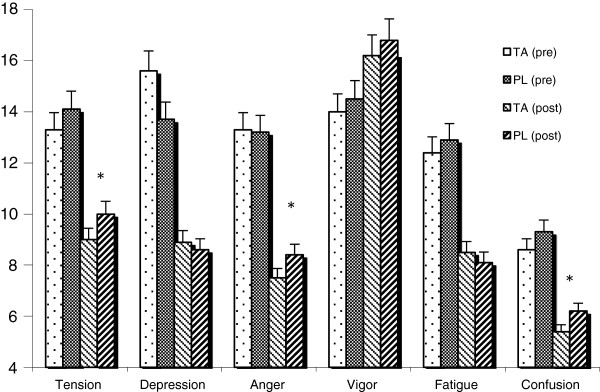
**Profile of Mood States (POMS).** Daily supplementation (200 mg/day for 4 weeks) with tongkat ali (TA) resulted in significant improvements compared to placebo (PL) for indices of Tension (−11%), Anger (−12%), and Confusion (−15%) in moderately stressed adults (N = 63). * = p < 0.05 by ANOVA.

Mood state parameters showed mixed results (Figure 1), with no effect observed between supplementation groups for indices of Depression, Vigor, or Fatigue, whereas significant improvements were found in the TA group for Tension (−11%), Anger (−12%), and Confusion (−15%) compared to placebo. A non-significant trend (p = .083) was found for an improvement in overall well-being in the TA group (+3% in Global Mood State).

**Figure 2:**
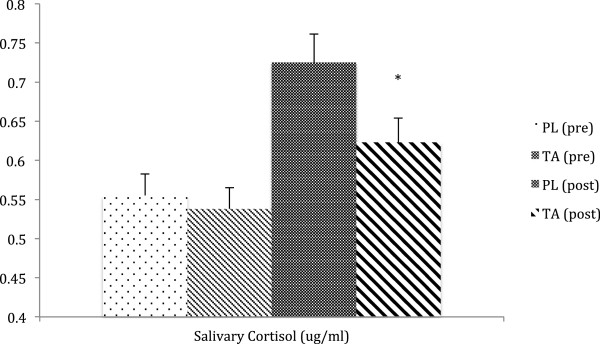
**Salivary cortisol.** Salivary cortisol levels were significantly lower (−16% compared to placebo, PL) following tongkat ali (TA) supplementation (200 mg/day for 4 weeks). * = p < 0.05 by ANOVA.

**Figure 3:**
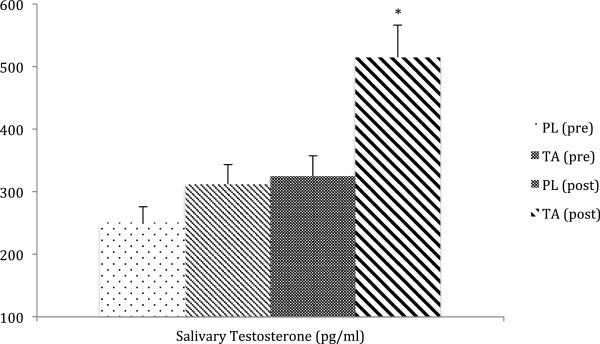
**Salivary testosterone.** Salivary testosterone levels were significantly higher (+37% compared to placebo, PL) following tongkat ali (TA) supplementation (200 mg/day for 4 weeks). * = p < 0.05 by ANOVA.

Hormone profile (salivary cortisol and testosterone) was significantly improved by TA supplementation, with reduced cortisol exposure (−16%, Figure 2), increased testosterone status (+37%, Figure 3) and overall improved cortisol:testosterone ratio (−36%) in the TA group compared to placebo.

## Discussion

The current study found that daily supplementation with tongkat ali root extract (200 mg/day) improves stress hormone profile (lower cortisol; higher testosterone) and certain mood state parameters (lower tension, anger, and confusion). These findings are in agreement with several recent supplementation trials in humans, suggesting that tongkat ali may be an effective approach to shielding the body from the detrimental effects of chronic stress from daily stressors, dieting for weight loss, sleep deprivation, and intense exercise training.

Previous studies have determined that *Eurycoma longifolia* contains a group of small peptides referred to as “eurypeptides” that are known to have effects in improving energy status and sex drive in studies of rodents [14-16]. The precise mechanism by which eurypeptides or tongkat ali root extract restores normal testosterone levels is unknown, but has been suggested as influencing the release rate of “free” testosterone from its binding hormone, sex-hormone-binding-globulin (SHBG) [17,18].

In two recent studies of young men undergoing a weight-training regimen [43,44] tongkat ali supplementation (100 mg/day) improved lean body mass, 1-RM strength, and arm circumference to a significantly greater degree compared to a placebo group.

In a recent 12-week trial [46] of *Eurycoma longifolia* supplementation (300 mg/day), men (30–55 years of age) showed significant improved compared to placebo in the Physical Functioning domain of the SF-36 quality of life survey. In addition, sexual libido was increased by 11% (week 6) and 14% (week 12) and abdominal fat mass was significantly reduced in subjects with BMI > 25 kg/m^2^.

In men with low testosterone levels (average age 51 years), one month of daily supplementation with tongkat ali extract (200 mg/day) resulted in a significant improvement in serum testosterone levels and quality-of-life parameters [41], suggesting a role for tongkat ali as an “adaptogen” against aging-related stress. Another study of healthy adult males (average age 25 years), 100 mg/day of tongkat ali extract added to an intensive strength training program (every other day for 8 weeks) resulted in significant improvements in fat-free mass, fat mass, maximal strength (1-RM) and arm circumference compared to a placebo group [43]. These results indicate that tongkat ali extract is able to enhance muscle mass and strength gains, while accelerating fat loss, in healthy exercisers, and thus, may be considered a natural ergogenic aid for athletes and dieters alike.

One study of middle-aged women (aged 45–59 years) found that twice-weekly strength training plus 100 mg/day of *Eurycoma longifolia* extract for 12 weeks enhanced fat free mass to a greater degree compared to women adhering to the same strength training program and taking a placebo [44]. Additional studies in dieters [48-50] and athletes [47] have shown 50-100 mg/day of tongkat ali extract to help restore normal testosterone levels in supplemented dieters (compared to a typical drop in testosterone among non-supplemented dieters) and supplemented athletes (compared to a typical drop in non-supplemented athletes). In one trial of endurance cyclists [47] cortisol levels were 32% lower and testosterone levels were 16% higher in supplemented subjects compared to placebo, indicating a more favorable biochemical profile for promoting an “anabolic” hormone state.

For a dieter, it would be expected for cortisol to rise and testosterone to fall following several weeks of dieting [54]. This change in hormone balance (elevated cortisol and suppressed testosterone) is an important factor leading to the familiar “plateau” that many dieters hit (when weight loss slows/stops) after 6–8 weeks on a weight loss regimen. By maintaining normal testosterone levels, a dieter could expect to also maintain their muscle mass and metabolic rate (*versus* a drop in both subsequent to lower testosterone levels) – and thus continue to lose weight without plateauing.

For an athlete, the same rise in cortisol and drop in testosterone is an early signal of “overtraining” – a syndrome characterized by reduced performance, increased injury rates, suppressed immune system activity, increased appetite, moodiness, and weight gain [55]. Maintenance of normal cortisol/testosterone levels in eurycoma-supplemented subjects may be able to prevent or reduce some of these overtraining symptoms as well as help the athlete to recover faster and more completely from daily training bouts.

These results indicate that daily supplementation with a properly standardized tongkat ali root extract improves stress hormone profile and certain mood state parameters, suggesting that this “ancient” remedy may be an effective approach to shielding the body from the detrimental effects of “modern” chronic stress, which may include general day-to-day stress, as well as the stress of dieting, sleep deprivation, and exercise training.

## Conclusions

A wide range of investigations, from laboratory research, to animal feeding studies, to human supplementation trials, have confirmed the health benefits and traditional use of tongkat ali root extract. Laboratory evidence shows that eurycoma peptides stimulate release of free testosterone from its binding proteins and improve overall hormone profiles. More than a dozen rodent feeding studies have demonstrated improved sex drive, balanced hormonal profiles, and enhanced physical function. Human supplementation trials show a clear indication of reduced fatigue, heightened energy and mood, and greater sense of well-being in subjects consuming tongkat ali root extracts. It is important to note that the majority of these studies, and all of the human supplementation trials, have been conducted on specific hot-water-extracts of *Eurycoma longifolia* (which is the traditional Malaysian preparation) produced using a patented extraction process to isolate and concentrate the bioactive compounds.

In conclusion, tongkat ali, used for centuries in traditional medicine systems of Southeast Asia for treating lethargy, low libido, depression, and fatigue, appears to have significant potential for restoring hormone balance (cortisol/testosterone) and improving psychological mood state in humans exposed to various modern stressors, including aging, dieting, and exercise stress.

## Competing interests

The authors have no directly competing interests, although one (AG) is an employee of a company that manufactures tongkat ali extract, and another (MP) is an employee of a nutrition company that uses tongkat ali as one ingredient in an anti-stress dietary supplement. The other authors (ST and JT) conducted this study as employees of SupplementWatch, which received funding for this trial from Biotropics Malaysia.

This study was funded by Biotropics Malaysia and conducted by SupplementWatch.

## Authors’ contributions

Each author contributed significantly to the successful carriage of this study. ST designed the study and drafted the manuscript. JT coordinated the IRB approval, subject visits, and sample inventory. AG and MP participated in the study design and coordination of subject visits. All authors read and approved the manuscript.
